# Antiproliferative and Apoptotic Effects of Olive Leaf Extract Microcapsules on MCF-7 and A549 Cancer Cells

**DOI:** 10.1021/acsomega.3c01493

**Published:** 2023-07-31

**Authors:** Yıldız Bal, Yusuf Sürmeli, Gülşah Şanlı-Mohamed

**Affiliations:** † Department of Biotechnology and Bioengineering, İzmir Institute of Technology, 35430 İzmir, Turkey; ‡ Department of Chemistry, İzmir Institute of Technology, 35430 İzmir, Turkey; § Department of Agricultural Biotechnology, Tekirdağ Namık Kemal University, 59030 Tekirdağ, Turkey

## Abstract

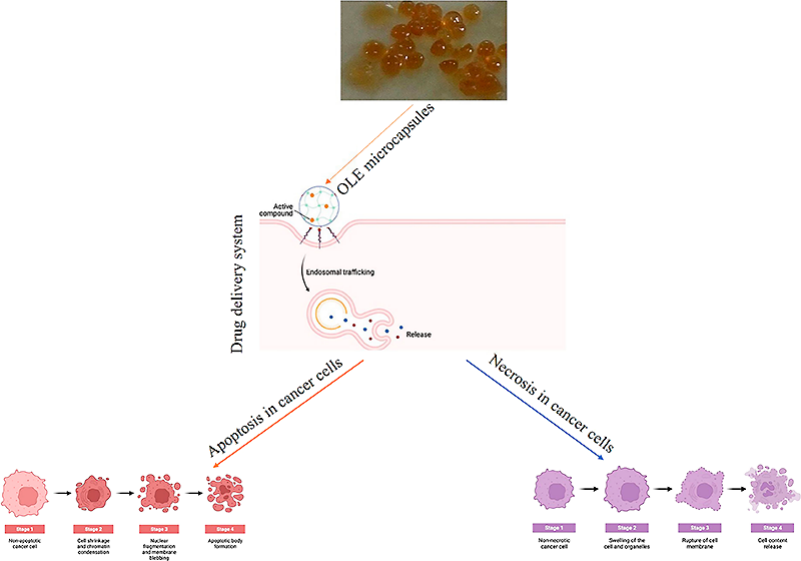

Alginate microcapsules are a talented means for the delivery of broad curative biomacromolecules. In this study, we immobilized olive leaf extract (OLE) by calcium alginate (CA) and chitosan-coated CA (CCA) and characterized the OLE-loaded CA and CCA. The cytotoxic effect, the cell cycle arrest, and the apoptotic effect of OLE and its microcapsules were investigated against breast adenocarcinoma (MCF-7) and lung carcinoma (A549). As a result, the loading capacity of OLE–CA and OLE–CCA was found to be 80 and 99%, respectively, in optimal conditions. Also, OLE–CA and OLE–CCA were characterized by unique FTIR peaks and morphological display relative to the empty CCA microcapsules. The cytotoxicity analysis showed that the IC_50_ values of OLE–CA and OLE–CCA were determined to be 312 and 0.94 μg mL^–1^ against A549, respectively, whereas these were found to be 865.4 and 425.5 μg mL^–1^ for MCF-7 cells. On the other hand, the OLE microcapsules did not possess in any concentration of cytotoxic influence on the BEAS 2B healthy cell line. Also, the exposure of OLE–CCA to MCF-7 and A549 resulted in the arrest of more MCF-7 and A549 cells at the G0/G1 phase compared to the OLE. A549 and MCF-7 cells were predominantly found in the late apoptosis phase and necrosis phase, respectively. Optical microscopy images confirmed that OLE microcapsules were more effective against MCF-7 and A549 than free OLE. The present work suggested that the OLE microcapsules might be administered as nutrition supplements for cancer therapy.

## 1 Introduction

Cancer is a highly important human disease worldwide, and it is the main cause of death following cardiovascular disorders.^1^ World Health Organization data predicts that approximately 30 million cancer cases will be reached by 2050.^2^ Chemotherapy drugs having cytotoxic effects are the gold standard for cancer cure.^3^ These drugs clearly impair the normal and cancer cells with rapid proliferation.^4^ Nevertheless, typical chemotherapy often results in unfavorable impacts including problems in the gastrointestinal system,^5^ toxic effects on the neural system,^6^ and kidneys.^7^ These drugs are continuously utilized for curing the various cancer types such as breast, prostate, lung, and colon. This highlights the significance of ongoing drug research of cancer treatment.^1^

Herbal medicines have been evaluated as alternative drugs for several centuries. Many plants and their substances have been reported on helpful curing impacts for various diseases like cancer.^8^ Among these plants, the olive tree (*Olea europaea*) has been utilized as a conventional plant-originated medicine in Mediterranean and European countries for many years.^9^ Olive leaves are the structures with anticancer features and other pharmaceutical characteristics such as antioxidant, antiviral (including SARS-CoV-2), antibacterial, anti-inflammatory, antidiabetic, neuroprotective, cardioprotective, hepatoprotective, and hypoglycemic.^10−12^ Olive leaf extract (OLE) is constituted by an elevated level of phenolic substances including apigenin, oleuropein, and hydroxytyrosol.^11^ It is accepted that phenolics are rapidly oxidized. This causes nigrescence, undesired smell, unpleasant taste, and loss of their effectiveness. Thus, they should be masked before their addition into foodstuffs or oral drugs.^13^

For several decades, drug delivery systems (DDSs) have been of great interest and have had an important place in the pharmaceutical industry. Researchers are investigating various methods of delivering therapeutic substances through different administration routes, with a focus on intricate dosage forms such as nanoparticles (liposomes, polymeric nanoparticles, and metallic nanoparticles), microspheres, transdermal DDSs, and mucoadhesive DDSs.^14−16^ These formulations offer benefits by delivering delicate molecules to the desired location through passive mechanisms (increased permeation and retention impact^17^) and/or active targeting (utilizing highly expressed receptors on diseased cells^18^). Additionally, they provide protection to the active molecules against degradation. For instance, alginate chitosan microspheres can shield it from the acidic gastric environment.^19,20^

Alginate microspheres are a talented means for the delivery of broad curative biomacromolecules.^21^ Alginate, consisting of repetitive units of 1–4 α-l-guluronic and β-d-mannuronic acid, is a water-soluble and naturally occurring biopolymer obtained from brown algae.^22^ The alginate polymer generates a hydrogel under the condition of bivalent cations including Ca^2+^, Zn^2+^, Sr^2+^, and Ba^2+^. This property of the alginate enables the preparation of drug-immobilized beads. Many reports have focused on the formation of calcium–alginate beads for controlled DDSs.^23^

The alginate can be adapted by various composites including chitosan and thus can form robust complexes for many applications.^21^ Chitosan, which can be obtained from the chitin of crustacean shells, consists of an unbranched polysaccharide structure of d-glucosamine and *N*-acetyl-d-glucosamine residues with the connection of β-(1–4) glycosidic linkages. Also, chitosan is a nontoxic, biologically degradable, and compatible substance.^24^ Hydrogels with the cross-linked alginate and chitosan polyelectrolyte complex are utilized to obtain favorable materials in pharmaceutical and medical uses and provide an increase in stability relative to the usage of the single polymer.^25^ In the current years, alginate–chitosan hydrogels have drawn great attention in controlled DDSs.^26^

Some works have been reported on hydrogels including the calcium alginate (CA) and chitosan–alginate polyelectrolyte complex to immobilize various therapeutic agents as reviewed in Dhamecha et al. (2019).^27^ In the literature, to the best of our knowledge, no work has been documented about OLE-loaded CA and chitosan-coated CA (CCA) so far. Thus, we studied the immobilization of OLE with CA and CCA, characterization of OLE–CA and OLE–CCA by Fourier transform infrared spectroscopy (FTIR), scanning electron microscopy (SEM), and optical microscopy and the investigation of the influence of OLE–CA and OLE–CCA on breast adenocarcinoma (MCF-7) and lung carcinoma (A549). This study offers that the OLE microcapsules might be administered as nutrition supplements for cancer therapy.

## 2 Materials and Methods

### 2.1 Materials

Unless otherwise stated, all chemicals were purchased from Sigma.

### 2.2 Preparation of OLE

OLE was prepared using olive leaves obtained from Olive Research Institute, İzmir, Turkey. For this purpose, after olive leaves were washed by dH_2_O, they were dried at 37 °C for 72 h. The dried leaves were then ground into powder and extracted using ethanol (70%) at 25 °C upon 2 h of incubation. The liquid phase was discarded by a vacuum filter, and ethanol was removed at 38 °C and 120 rpm using a rotary evaporator. OLE was dried by a freeze-dryer under −52 °C and 0.2 mbar conditions (Figure S1). It was stored in a light-protected bottle.

### 2.3 Characterization of OLE

#### 2.3.1 Total Phenolic Content in OLE

The total phenolic content (TPC) in OLE was determined via the Folin–Ciocalteu method previously described by Bayçın et al.^28^ For this purpose, the OLE was diluted using distilled water and gently vortexed. 20 μL of the sample was supplemented into a 96-well plate including 100 μL of the Folin reagent at room temperature (RT) for 2 min. 80 μL of Na_2_CO_3_ (7%) was supplemented to each sample, thoroughly mixed, and incubated for 1 h. The absorbance of the samples was spectrophotometrically measured at 725 nm. The analysis was performed in three biological repetitions, and the results were expressed as milligrams of gallic acid equivalents per gram of dry olive leaf (mg GAE g^–1^) using a gallic acid calibration curve (Figure S2).

#### 2.3.2 Determination of OLE Composition

To determine the OLE composition, high-performance liquid chromatography (HPLC) analysis was performed using a Hewlett-Packard Series HP 1100 equipped with a diode array detector and a C18 LiChrospher 100 analytical column (250 × 4 mm). The flow rate was adjusted to 1 mL min^–1^, and the change of absorbance was displayed at 280 nm wavelength. The mobile phases were used as 2.5/97.5 (v v^–1^) of acetic acid/water (A) and acetonitrile (B). A linear gradient was run for 60 min in total, from 95% A–5% B to 75% A–25% B for 20 min, and then it was changed into 50% A–50% B for 20 min and 20% A–80% B for 10 min, respectively. At last, re-equilibration was performed for 10 min to have the initial composition.

The oleuropein level in OLE was also determined by HPLC analysis using an oleuropein calibration curve (Figure S3). As an internal standard, coumarin was utilized for the measurement of antioxidant oleuropein.

#### 2.3.3 Total Antioxidant Capacity Analysis

Trolox equivalent antioxidant capacity (TEAC) analysis, based on scavenging of the radical cation 2,2′-azinobis-(3-ethylbenzothiazoline-6-sulfonic acid) (ABTS+) to convert it as a colorless product, was performed to determine the total antioxidant capacity. For this purpose, sodium persulfate (K_2_S_2_O_4_) was supplemented into ABTS + working solution at a ratio of 1:1 and incubated at RT in the dark for 12–16 h. The solution was measured at 734 nm by a spectrophotometer (Thermo, Varioskan Flash, U.S.A.), compared with Trolox, vitamin E analogue soluble in water, as a standard. The results were expressed as mmol TEAC/g OLE of the sample using a Trolox calibration curve (Figure S4).

### 2.4 Encapsulation of OLE by the CA Microcapsules

Encapsulation of OLE by CA and CCA microcapsules was simultaneously performed. For this purpose, 1.5% OLE was resuspended in the alginate solution (3% sodium alginate in 0.3 M Tris–HCl buffer (pH 8.5)). The solution was added to 1.7 M calcium chloride by a syringe to form CA beads. The beads were incubated to harden in a gelling bath for 60 min and then filtered. The OLE–CA microcapsules were immersed in the chitosan (medium *M*_w_ 480 kDa) solution for 30 min to get OLE–CCA microcapsules.

### 2.5 Optimization of the OLE–CA and OLE–CCA Microcapsules

The optimization of the OLE microcapsules was performed to examine the effect of various parameters including the concentrations of alginate, CaCl_2_, Tris–HCl buffer, chitosan, and OLE as well as the pH on loading capacity and proper round-shaped microcapsules. To do this, the microcapsules were obtained using alginate concentrations (1, 2, 3, and 4%), CaCl_2_ concentrations (1, 1.2, 1.4, 1.7, and 2 M), Tris–HCl concentrations (0.3, 0.5, 0.7, and 1 M), Tris–HCl pH values (pH 8, 8.5, and 9), chitosan concentrations (0.5, 1, 1.5, and 2%), and OLE concentrations (1.5, 2, and 3%).

### 2.6 Characterization of the OLE–CA and OLE–CCA Microcapsules

The loading capacity of the OLE–CA and OLE–CCA microcapsules was investigated by using the Folin–Ciocalteu method.^29^ For this purpose, OLE–CA and OLE–CCA microcapsules were resuspended into the 10% Na–citrate solution at 37 °C and 125 rpm for 20 and 60 min, respectively. The supernatant was spectrophotometrically measured at 750 nm to calculate the loading capacity. The percent loading capacity was expressed using the following equation
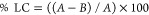
1where *A* is the initial amount of OLE in the alginate solution and *B* is the amount of OLE in the sodium citrate solution.

The interactions between OLE and CCA were investigated using OLE–CCA and empty CCA by FTIR. To do this, the Miracle Zn–Se ATR method was performed on a Spectrum-100 FT-IR Spectrometer (PerkinElmer) in the range of 650–4000 cm^–1^. In addition, the investigation of the geometry of the empty CCA and OLE–CCA beads was performed by using optical microscopy (OLYMPUS-CKX41). The external morphology of the beads was investigated by a scanning electron microscope (Quanta 250) in the environmental SEM (ESEM) mode, allowing the study on moist microcapsules.

### 2.7 Effect of OLE Microcapsules on Cancer Cell Lines

#### 2.7.1 In Vitro Cytotoxicity Assay

In vitro cytotoxicity analysis was performed to analyze the cytotoxic impacts of different concentrations of OLE–CA and OLE–CCA microcapsules against MCF-7 (breast adenocarcinoma), BEAS 2B (human bronchial epithelium), and A549 (lung carcinoma) by using the MTT method. For this purpose, MCF-7, A549, and BEAS 2B were cultured into 96-well plates using 100 μL of 1 × 10^4^ cells mL^–1^ upon incubation of 24 h. MCF-7, A549, and BEAS 2B cells were then exposed to free OLE and OLE microcapsules in the range of 1–1000, 200–1000, and 200–1000 μg, respectively. The treated cells, as well as untreated (control) cells, were incubated at 37 °C for 72 h. The cells were then washed using phosphate-buffered saline (PBS) buffer. 100 μL of 10% MTT was supplemented into each well, and the incubation of the plates were performed under 37°C–5% CO_2_ condition for 4 h. The cultures were harvested at 1800 rpm to 10 min, and the pellets were resuspended with dimethyl sulfoxide (DMSO). The absorbance was measured at 540 nm. The cell viability was calculated using the following equation
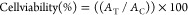
2where *A*_C_ and *A*_T_ are the absorbance values for the control and treated cells, respectively.

IC_50_ was determined by using cell viability curves. This analysis was performed as three biological repetitions.

#### 2.7.2 Cell Cycle Analysis

The antiproliferative activity of free OLE and OLE–CCA on MCF-7 and A549 cell cycles was analyzed by flow cytometry using propidium iodide (PI) staining as the fluorescent staining. To do this, 1 × 10^5^ cells of A549 and MCF-7 were cultured into six-well plates including 1.98 mL of growth medium and incubated overnight. 20 μL of free OLE and OLE–CCA microcapsules was supplemented into each cell line to a final concentration of 50, 100, 500, and 1000 μg mL^–1^. The treated and untreated (control) cells were incubated under 37°C–5% CO_2_ condition for 72 h. Then, the cells were washed with PBS and harvested by trypsinization. Then, 4 mL of cold ethanol was gently added to the cell suspensions for cell fixation on ice. The fixed cells were centrifuged at 4 °C and 1200*g* for 10 min. The cell pellets were then dissolved in 200 μL of Triton X-100 (0.1%), and 20 μL of RNase A (200 μg mL^–1^) was supplemented to cell suspensions and then incubated under 37 °C and 5% CO_2_ conditions for 30 min. 20 μL of 1 mg mL^–1^ PI was added, and the samples were incubated at RT for 15 min. The cell cycle distribution was detected using a flow cytometer (FACSCANTO, BD), and data analysis was performed using ModFit software to collect at least 10.000 events for each sample.

#### 2.7.3 Apoptosis Analysis

The apoptosis analysis was performed for investigating the effect of the OLE–CCA microcapsules on A549 and MCF-7 cell lines by the Annexin V-FITC Detection Kit. 1 × 10^5^ cells were cultured using a six-well plate including 1.98 mL of growth medium and incubated under 37°C–5% CO_2_ condition for 24 h. 20 μL of the OLE–CCA microcapsules was then supplemented in the range of 50–1000 μg mL^–1^ concentrations. The treated and untreated (control) cells were incubated under 37 °C–5% CO_2_ condition for 48 h. The cells were centrifuged twice at 800 rpm for 5 min and washed using PBS. The pellets were dissolved in 200 μL of binding buffer. Then, 2 μL of Annexin V-FITC and PI was added into each sample. The stained cells were incubated at RT for 15 min and tested by a flow cytometer (FACSCANTO, BD).

#### 2.7.4 Optical Microscopy Analysis

The effects of the free OLE and OLE–CCA microcapsules were visually investigated by optical microscopy. To do this, MCF-7 and A549 cells were cultured using a 96-well plate. Free OLE, OLE–CA, and OLE–CCA microcapsules were supplemented into each well including the cell lines at the cytotoxic dose and incubated for 72 h. The treated and untreated (control) cells were displayed using optical microscopy (OLYMPUS-CKX41).

## 3 Results and Discussion

In the present work, OLE was immobilized by CA and CCA microcapsules. The formation of OLE–CA and OLE–CCA microcapsules was optimized by investigation of different concentrations of alginate, chitosan, Tris–HCl, calcium chloride, and OLE, as well as different pH values of Tris–HCl buffer. Then, OLE–CA and OLE–CCA microcapsules were characterized by FTIR and morphological analyses (optical microscopy and SEM). The effect of OLE–CA and OLE–CCA was investigated on two types of cancer cell lines (breast adenocarcinoma MCF-7 and lung carcinoma A549) and human bronchial epithelium cell lines (BEAS 2B). For this purpose, OLE was prepared and characterized to determine its TPC, OLE composition, and total antioxidant capacity. TPC in OLE was determined by the Folin–Ciocalteu method. The analysis result showed that the level of the total phenolic compound was found to be 0.26 mg mL^–1^ and was calculated as 260 mg GAE g^–1^ extract. Also, the OLE composition was determined by HPLC analysis (Figure S5). As a result of this analysis, oleuropein was found as the most abundant phenolic substance in OLE among other substances and determined to be 0.23 μg mL^–1^ with 2.3% (w v^–1^) of OLE. Many studies have been reported which showed that oleuropein was an anticancer agent as reviewed in Farooqi et al. (2017).^30^ Interestingly, it has been demonstrated that this phenolic compound was also effective against SARS-CoV-2. leading to the Coronavirus disease (Covid-19).^12^ In addition, the total antioxidant capacity of OLE was investigated by the TEAC assay. The results indicated that the total antioxidant capacity of OLE was 2.18 mmol of TEAC g^–1^ OLE.

### 3.1 Optimization of OLE–CA and OLE–CCA Microcapsules

**Figure 1:**
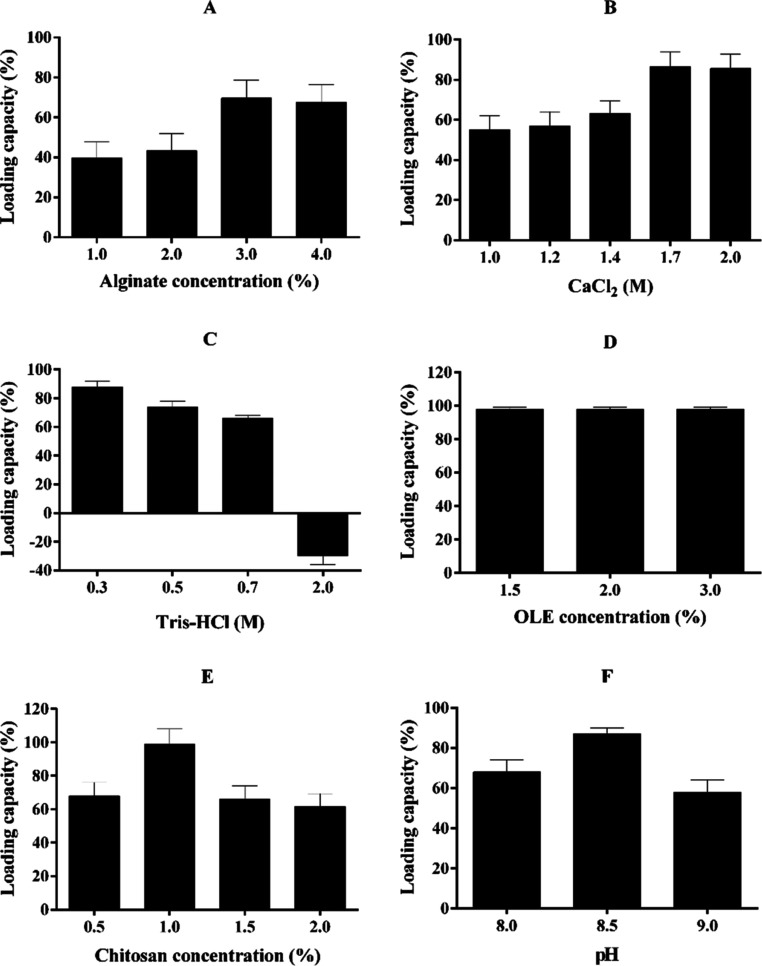
Optimization of OLE–CCA microcapsules at various concentrations of alginate (A) concentration, CaCl_2_ concentration (B), Tris–HCl buffer concentration (C), OLE concentration (D), chitosan concentration (E), and pH of Tris–HCl (F).

OLE was immobilized by CA and CCA microcapsules. Then, the OLE-loaded microcapsules were optimized by investigating the effects of alginate concentration (1, 2, 3, and 4%), CaCl_2_ concentration (1, 1.2, 1.4, 1.7, and 2 M), Tris–HCl buffer concentration (0.3, 0.5, 0.7, and 1 M), pH (pH 8.0, 8.5, and 9.0), chitosan concentration (0.5, 1, 1.5, and 2%), and OLE concentration (1.5, 2, and 3%) on loading capacity and microcapsule formation. In effect of different alginate concentrations, the highest loading capacity of OLE–CA was determined at a 3% alginate concentration by 68%. There was no difference in loading capacity between 3 and 4% alginate concentrations. The loading capacities at these concentrations were significantly higher than those of 1 and 2% alginate concentrations (Figure 1A). Also, the optimum loading capacity in both OLE–CA and OLE–CCA microcapsules was observed at 1.7 M CaCl_2_. Here, the two types of encapsulated OLE exhibited similar loading capacity profiles. OLE–CCA microcapsules had higher loading capacity than the OLE–CA in the presence of all CaCl_2_ concentrations, except 2 M (Figure 1B). In addition, the highest loading capacity of OLE-CA was found to be 88 and 87% at 0.3 M and pH 8.5 Tris–HCl buffer, respectively (Figure 1C,F). Besides this, no differences were found between OLE concentrations (1.5, 2, and 3%) for OLE–CA (Figure 1D); however, the smoothest round shape of OLE-loaded microcapsules was found at 1.5% OLE (Figure S6). Also, the optimum chitosan concentration for OLE–CA was found to be 1%, compared to the other concentrations (Figure 1E). Further analyses were performed at optimum conditions as 3% alginate, 1.7 M CaCl_2_, 0.3 M Tris–HCl (pH 8.5), 1% chitosan, and 1.5% OLE.

### 3.2 Characterization of OLE–CA and OLE–CCA

**Figure 2:**
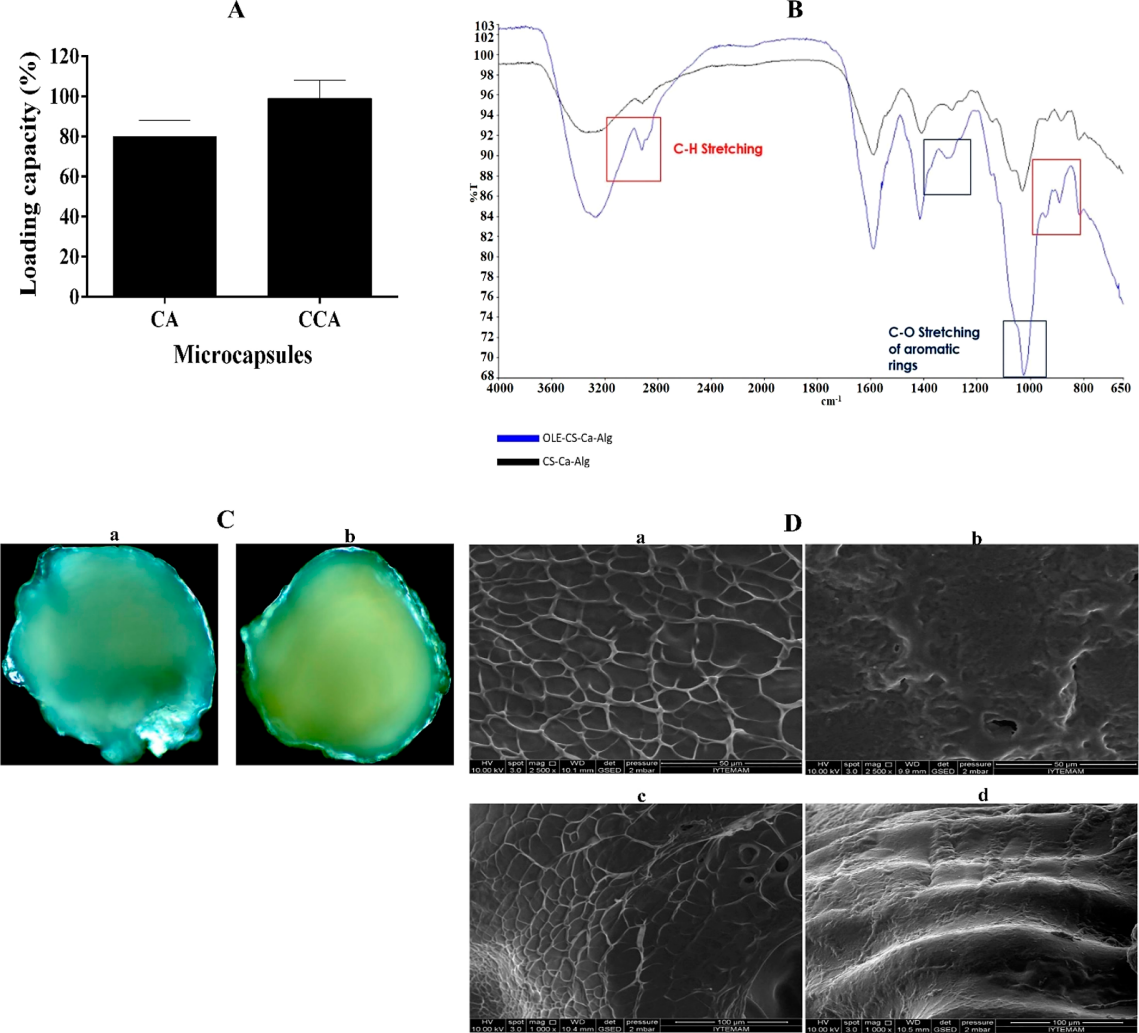
Characterization of the encapsulated OLE by calcium–alginate (OLE–CA) and/or chitosan-coated calcium–alginate (OLE–CCA) (A) loading capacity of CA and CCA at optimum conditions, (B) FTIR spectra of OLE–CCA and empty capsule CCA, (C) optical microscopy images of microcapsules CCA (a) and OLE–CCA (b), and (D) ESEM image of empty CCA (a,c) and OLE–CCA (b,d) microcapsules at 2500× and 1000× magnification, respectively.

OLE–CA and OLE–CCA were characterized by investigating the loading capacity, interactions between OLE and CCA, and morphology. The loading capacity of OLE–CA and OLE–CCA was determined by the Folin–Ciocalteu method, a spectrophotometric analysis. The result showed that OLE–CA and OLE–CCA microcapsules had a loading capacity of about 80 and 100%, respectively (Figure 2A). These results indicated that CA encapsulation was a powerful approach for OLE immobilization. Also, the interactions between OLE and CCA in OLE–CCA were investigated by FTIR spectroscopy, compared to the CCA without OLE (Figure 2B). The FTIR spectra results showed that the peaks in some regions 3200–2800, 1400–1200, and 1000–800 cm^–1^ of OLE–CCA were sharper than that of the empty CCA microcapsules. The peaks between 3000 and 2800 cm^–1^ might be attributed to the stretching NH_2_ functional group, which overlapped with the OH peak found in the range of 3000–3600 cm^–1^. Also, OLE–CCA capsules exhibited a tiny peak in the interval of 2800–3200 and 1650–1700 cm^–1^; however, empty microcapsules did not have those peaks (Figure 2B). These interactions might be unique to the CCA encapsulation-based systems. Morphological analysis of OLE–CCA was investigated by optical microscopy and ESEM at magnitudes of 2500× and 1000×, relative to the empty CCA capsules. Optical microscopy analysis indicated that both capsules are round-shaped having some aggregations (Figure 2C). According to the ESEM analysis, the results showed that CCA capsules have rough surface in a spider-net like structure containing a various sized holes. OLE–CCA microcapsules had a smoother surface, relative to the empty CCA capsules, due to the possible effect of phenolic compounds (Figure 2D).

### 3.3 Investigation of the Effect of OLE Microcapsules on Cancer Cell Lines

#### 3.3.1 In Vitro Cytotoxicity Analysis

**Figure 3:**
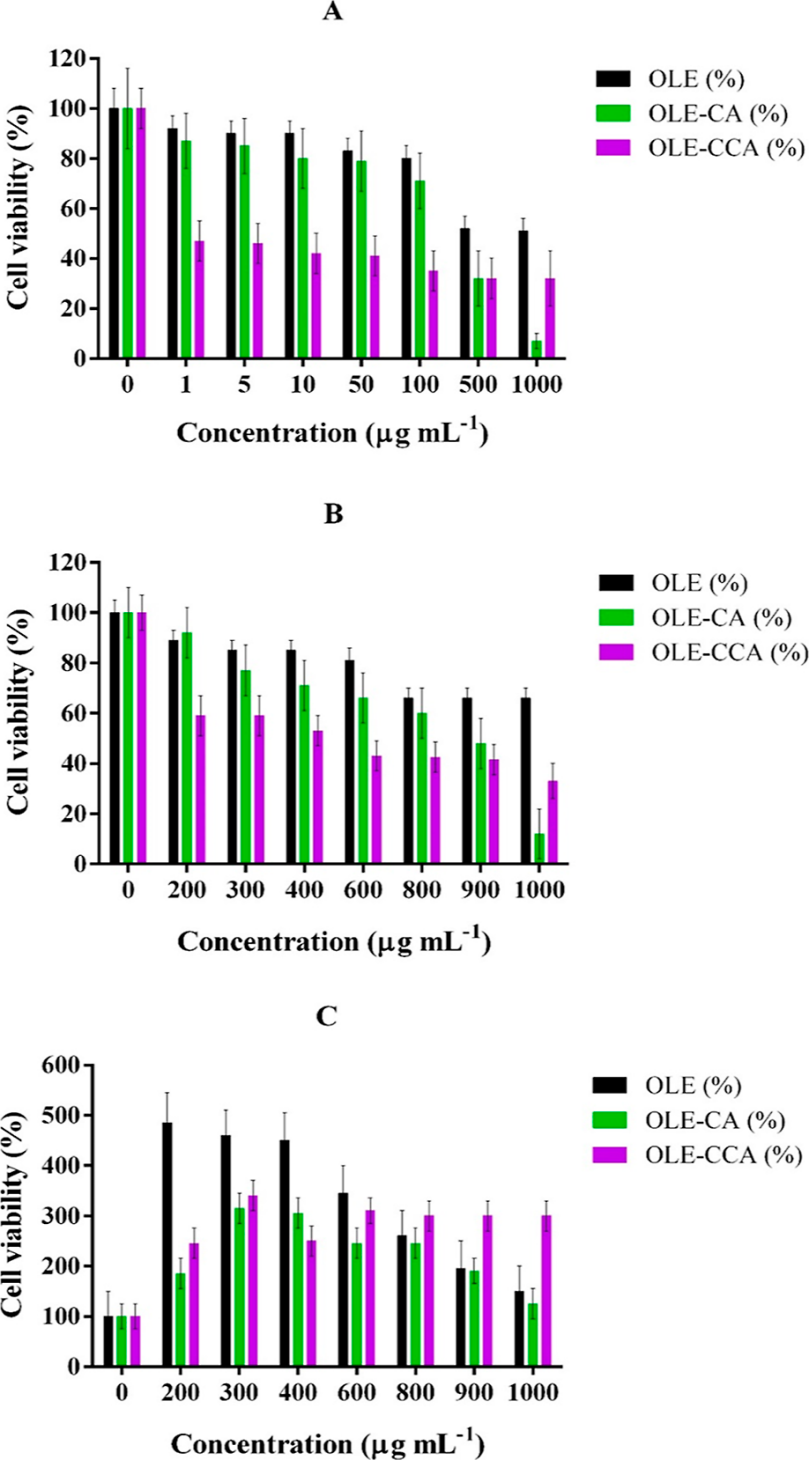
Cytotoxic effect of OLE, OLE–CA, and OLE–CCA on A549 (A), MCF-7 (B), and BEAS 2B (C) cell lines.

The cytotoxic effect of different concentrations of OLE–CCA, OLE–CA, OLE, CCA, alginate, and chitosan on two different cancer cell lines (A549 and MCF-7), as well as the healthy cell line (BEAS 2B), was analyzed by MTT analysis and is summarized in Figure 3 and Table 1. The analysis results showed that the cell viability of A549 and MCF-7 was found to be 51 and 66% at 1000 μg mL^–1^ free OLE, respectively. It is well known that olive leaves and their phenolics including oleuropein had a cytotoxic effect on different cancer cell types including A549 and MCF-7.^31−35^ In the present study, OLE–CA and OLE–CCA significantly decreased the viable cell number of A549 and MCF-7. Regarding this, it was found to be 7 and 32% of A549 cell viability for OLE–CA and OLE–CCA, respectively, at 1000 μg mL^–1^ (Figure 3A), whereas these values were determined to be 12 and 33% for the MCF-7 cell line (Figure 3B). The IC_50_ value of OLE-CA was found to be 312 μg mL^–1^ for A549 and 865.4 μg mL^–1^ for MCF-7, whereas the IC_50_ value of OLE–CCA was found to be 0.94 and 425.5 μg mL^–1^ for A549 and MCF7, respectively. Compared to the OLE–CA capsules, OLE–CCA encapsulation had higher sensitivity against A549 and MCF-7 cells. OLE–CCA exhibited the highest cytotoxicity against A549, relative to MCF-7 (Table 1).

**Table 1:** IC_50_ Values of OLE–CA and OLE–CCA against A549 and MCF-7

cancer cell type	OLE–CA (μg mL^–1^)	OLE–CCA (μg mL^–1^)
A549	312	0.94
MCF-7	865.4	425.5

No significant toxic effects of alginate, chitosan, and empty CCA capsules against cancer cell lines were observed even at the highest concentration tested. We presumed that the effect of chitosan could be varied among cancer cell lines. Thus, we might observe the obvious positive effect of chitosan on A549, relative to MCF-7 (Figure S7). In addition, OLE–CA and OLE–CCA microcapsules and free OLE highly stimulate the cell proliferation of the BEAS 2B healthy cell line up to 1000 μg mL^–1^. BEAS 2B displayed a distinct pattern compared to A549 and MCF-7 when exposed to OLE, OLE–CA, and OLE–CCA. We noted that they might have a dose-dependent effect and/or mitotic index effect on BEAS 2B. At lower doses, a significantly higher proliferation was observed. However, as the dosage increased, the extent of this proliferation declined (Figure 3C). We anticipated that this proliferation effect may further decrease at concentrations exceeding 1000 μg mL^–1^, potentially leading to cytotoxic effects with cell viability dropping below 100%.

There have been limited studies on the effect of the immobilized OLE against the cancer cell types. Accordingly, the immobilization with silver nanoparticles (AgNPs) led to the decrease of MCF-7 viability up to 62% at 50 μg mL^–1^.^36^ Several studies in the literature have explored the cytotoxic effects of diverse drugs loaded into CA beads against various types of cancer cells, highlighting the potential benefits of these novel materials. These studies provide valuable evidence of the effectiveness and practical applications of drug-loaded CA beads in the field of cancer research. Regarding this, Ca-alginate apigenin-loaded beads (BD6) significantly decreased MCF-7 cell survival, compared to pure apigenin (AGN).^37^ The same study showed that the encapsulation of AGN with Ca-alginate has led to a slight increase of IC_50_ against MCF-7 cells.^37^ In another study, Wiwattanapatapee et al. (2015) developed CA beads containing curcumin (SE-Cur) for targeted delivery to the colon, achieving an IC50 value of 10 μg/mL.^38^ The SE-Cur-loaded alginate beads demonstrated improved activity and played a crucial role in delivering poorly soluble drugs to the colon. Furthermore, alginate beads were utilized for the formulation of cetuximab (CTX) drug (CTX-OCT-Alg) to facilitate targeted oral drug delivery, minimizing off-site targets and reducing side effects. Additionally, this approach helped lower the dosage of the anticancer agent required for therapeutic response.^39^ The study revealed that CTX-OCT-Alg beads exhibited excellent gastroresistant properties and efficiently delivered anticancer drugs to the higher pH environments of the colon, resulting in higher antiproliferative activity compared to free drugs. Also, the pure drug (roflumilast) had a 2.5 time higher IC_50_ value than that for drug-loaded CD microparticles against the A549 cell line.^40^ In our study, OLE–CA led to the decrease of IC_50_ value by at least 3.5- and 1.5-fold against A549 and MCF-7, respectively, compared to the free OLE. On the other hand, OLE–CCA resulted in the decrease of IC_50_ by 100 times and 2.5 times against A549 and MCF-7, respectively (Table 1).

#### 3.3.2 Analysis of Cell Cycle Checkpoints of Cancer Cell Lines Treated by OLE and OLE–CCA

**Figure 4:**
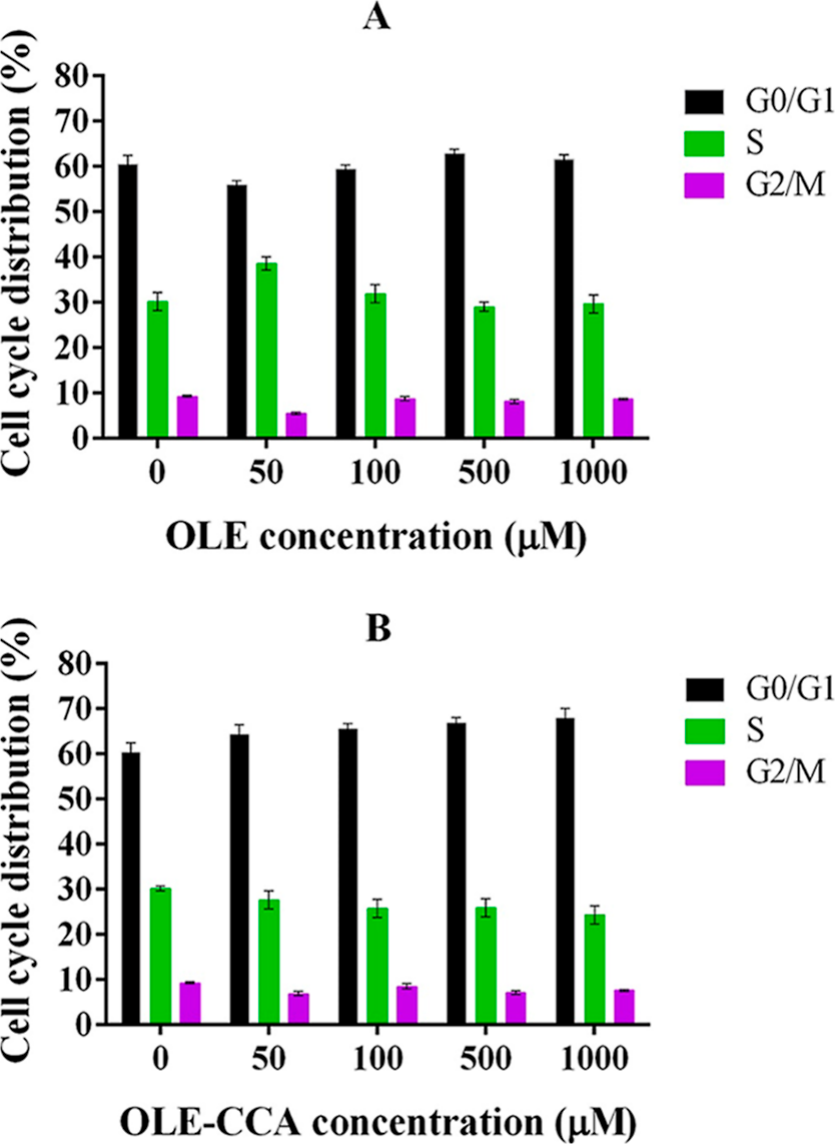
Effect of OLE (a) and OLE–CCA (b) on cell cycle arrest in A549 cells.

The cell cycle checkpoint distributions of A549 and MCF-7 were investigated using a flow cytometer by treatment with different concentrations (50, 100, 500, and 1000 μM) of free OLE and OLE–CCA microcapsule as well as untreated (control) cell lines. The results showed that both cell types exhibited a similar pattern against free OLE or OLE–CCA in terms of the percent of cell cycle arrest in G0/G1, S, and G2/M checkpoints. Accordingly, more than half of the cells were arrested at the G0/G1 checkpoint, and the smallest percent of the arrested cells was detected in the G2/M checkpoint when treated with the specified concentrations of OLE and OLE–CCA on A549 (Figure 4) and MCF-7 (Figure S8) cells. OLE arrested a higher number of A549 and MCF-7 cells than OLE–CCA microcapsules at the S phase (Figures 4A and S8A). In contrast, OLE–CCA arrested both cell types at a higher percent ratio of the G0/G1 checkpoint (Figures 4B and S8B). This indicated an increment in the cell number of two cell types in the G0/G1 phase. The ratio of the arrested cells at this G0/G1 checkpoint increased with the increasing concentration of OLE–CCA. Nevertheless, there was no considerable difference between untreated (control) and OLE-treated cells even at high concentrations. Taken together, OLE–CCA microcapsules had higher effectiveness on perturbation of the cell cycle of cancer cells (A549 and MCF-7), compared to the free OLE. There have been similar results in the literature about cell cycle distribution in MCF-7 cells. In one study, OLE inhibited a large number of MCF-7 cells at the G0/G1 phase compared to the other cell cycle checkpoints by decreasing the amount of cyclin D1 protein. Thus, this work suggests that further research would need for the possible utilization of OLE in cancer treatment as a food additive.^41^ Similarly, hydroxytyrosol blocked the cell cycle in G0/G1, reducing the level of G1 protein (cyclin D1).^42^ In addition, oleuropein and hydroxytyrosol found in OLE could halt the cell cycle in the G1 phase of MCF-7 cells by inducing apoptosis.^43^

#### 3.3.3 Apoptotic Effect of OLE–CCA on Cancer Cell Lines

**Figure 5:**
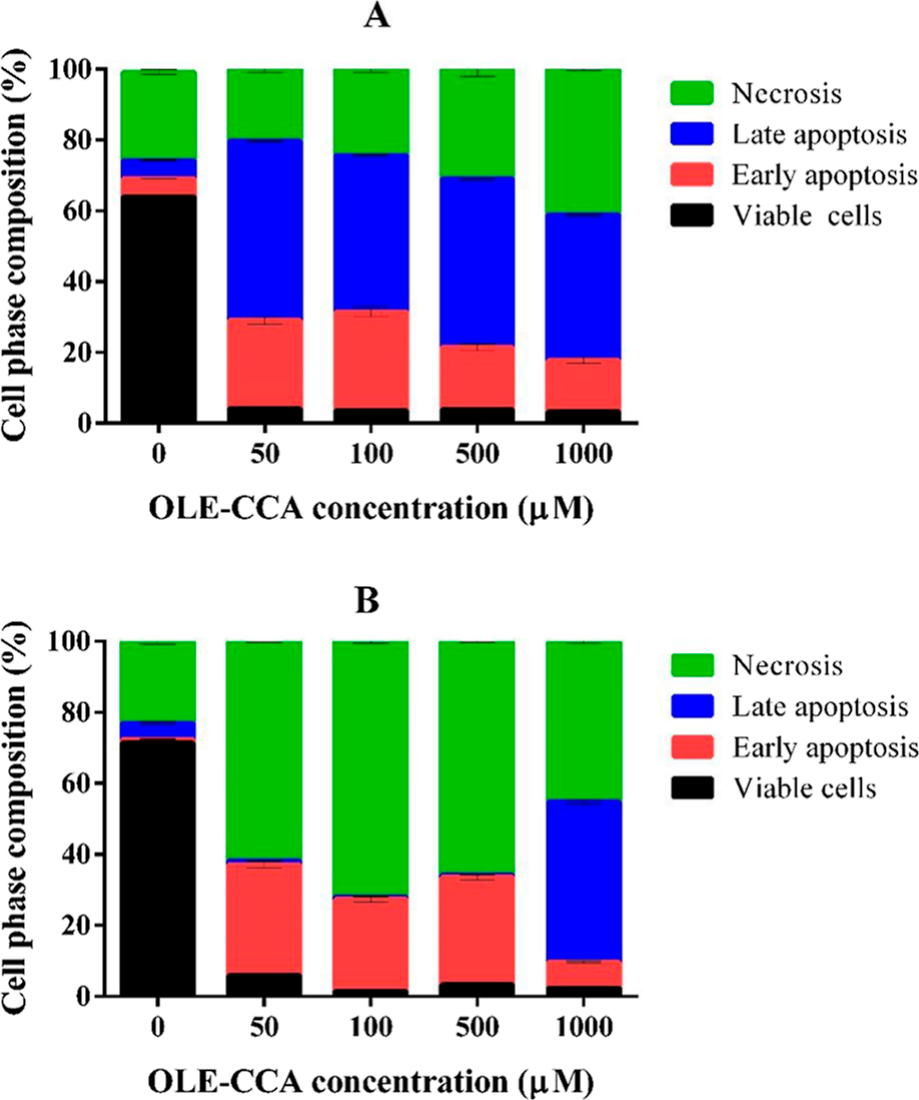
Apoptotic effect of OLE–CCA on A549 (A) and MCF-7 (B).

The apoptotic effects on lung carcinoma A549 cells and breast adenocarcinoma MCF-7 cells treated with various levels of OLE-CCA capsules (50, 100, 500, and 1000 μg mL^–1^), as well as untreated (control) cells, were investigated by the Annexin V/PI staining method. The results indicated that all the concentrations of OLE–CCA microcapsules significantly triggered the cell death of lung carcinoma A549 cells by a considerable decrease of viable cell number and an increase of cells in apoptosis phases (especially late phase). The cell number in the necrosis phase was significantly enhanced with the increasing concentrations of OLE–CCA, relative to the untreated (control) A549 cells (Figure 5A). All the concentrations of OLE–CCA held MCF-7 cells in the necrosis phase at the top level compared to the control (untreated) cells and decreased viable cells to a great extent. The MCF-7 cells in the early apoptosis phase were more than the control cells; however, the cells in this phase decreased with the increase of OLE–CCA concentrations. MCF-7 control cells in late apoptosis were slightly lower than OLE–CCA treated cells with the compound of 50, 100, and 500 μg mL^–1^. Interestingly, the cells in late apoptosis were at the top level when treated with 1000 μg mL^–1^ OLE–CCA (Figure 5B). Some works have been documented on the apoptotic effect of OLE-included oleuropein against A549 and MCF-7 cells. Regarding this, one study on MCF-7 cells showed that oleuropein promoted apoptosis through the mitochondrial pathway, whereas another study indicated that oleuropein stimulated apoptosis through modulating NF-kB activation cascade.^44,45^ On the other hand, one work showed that the oleuropein induced apoptosis by upregulating the mitochondrial protein Glo2 mediated by two factors (superoxide anion and Akt signaling pathway).^46^

**Figure 6:**
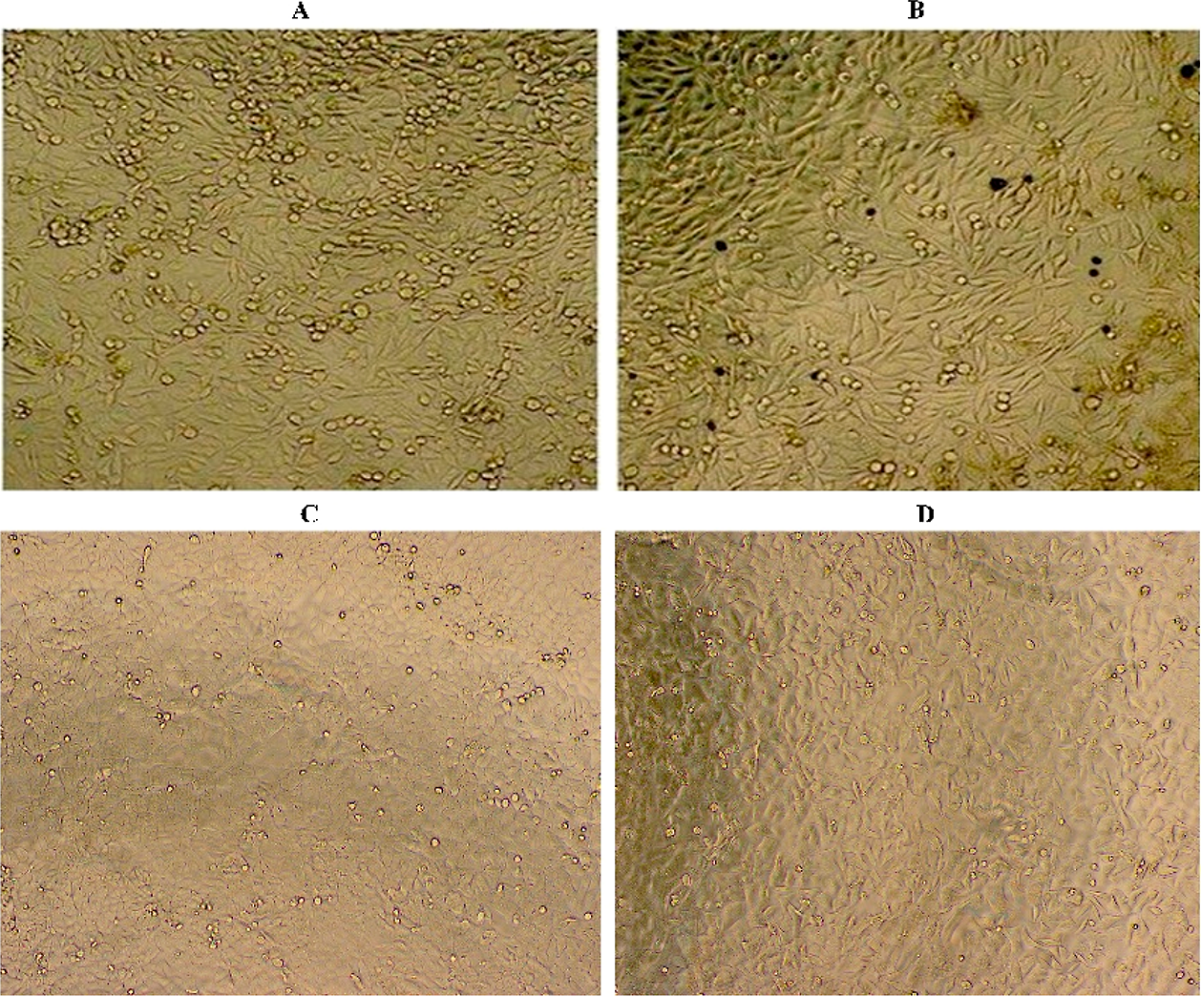
Optical microscopy images of A549 and MCF-7 cells treated with OLE–CCA (B,D) as well as untreated (control) cell lines (A,C), respectively.

According to the optical microscopy analyses, a reduction in cell number and significant morphological changes were monitored in the presence of OLE–CCA capsules, relative to the other conditions. Shrinking the cell and forming the apoptotic bodies were apparently seen in A549 and MCF-7 OLE–CCA-treated cells (Figure 6B,D). On the other hand, these morphological changes were not detected in untreated (control) cells (Figure 6A,C). These results supported in vitro cytotoxicity results.

## 4 Conclusions

In the present work, olive leaves from the olive tree *O. europaea* were extracted, immobilized, characterized, and investigated for their effects against breast adenocarcinoma MCF-7 and lung carcinoma A549. This study was the first report on the immobilization of OLE by CA and CCA. The analysis results showed that the loading capacity of CA and CCA microcapsules was found to be approximately 80 and 100%, respectively, under optimal conditions. In addition, cytotoxicity analysis demonstrated that OLE–CA and OLE–CCA possessed higher cytotoxic effects against breast adenocarcinoma MCF-7 and lung carcinoma A549, relative to the free OLE. Strikingly, OLE–CCA had great effectiveness on A549, exhibiting very low IC_50_ by 0.94 μg mL^–1^. Also, these OLE microcapsules did not have any cytotoxic effect on the BEAS 2B healthy cell line. The cell cycle distribution analysis results indicated that many of the A549 and MCF-7 cells treated with free OLE and OLE–CCA accumulated in the G0/G1 phase. Apoptosis analysis results showed that OLE–CCA was a potential anticancer agent causing cell death of A549 and MCF-7. Based on our results, the use of OLE microcapsules might be offered as supplements for cancer therapy.

## Supporting Information Available

Extraction of olive leaves; calibration curves of TPC, total antioxidant capacity; HPLC of OLE; increasing OLE concentration on capsule formation; and cytotoxic effect and cell cycle arrest (PDF)

## Notes

This article does not contain any studies with human participants or animals performed by any of the authors.
