# Supplementary material for: Antiproliferative and Apoptotic Effects of Olive Leaf Extract Microcapsules on MCF-7 and A549 Cancer Cells
Source: ACS Omega. 2023 Jul 31;8(32):28984–93. doi: 10.1021/acsomega.3c01493 (PMC10433482; doi:10.1021/acsomega.3c01493)
Supplement: Supplementary file 1 — ao3c01493_si_001.pdf [file ao3c01493_si_001.pdf]

Supporting Information

Anti-proliferative and Apoptotic Effects of Olive Leaf Extract Microcapsules on MCF-7 and A549 Cancer Cells

Yıldız Bal[1], Yusuf Sürmeli[1,3] and Gülşah Şanlı-Mohamed[*1,2]

1. Department of Biotechnology and Bioengineering, İzmir Institute of Technology, 35430, İzmir, Turkey
2. Department of Chemistry, İzmir Institute of Technology, 35430, İzmir, Turkey
3. Department of Agricultural Biotechnology, Tekirdağ Namık Kemal University, 59030, Tekirdağ, Turkey

[*]Corresponding Author

Prof. Dr. Gülşah Şanlı-Mohamed

Izmir Institute of Technology, Science Faculty, Department of Chemistry

Urla, Izmir, Turkey

Phone: +90 2327507515; Fax: +90 2327507509

E-mail: gulsahsanli@iyte.edu.tr, gulsahsanli@gmail.com

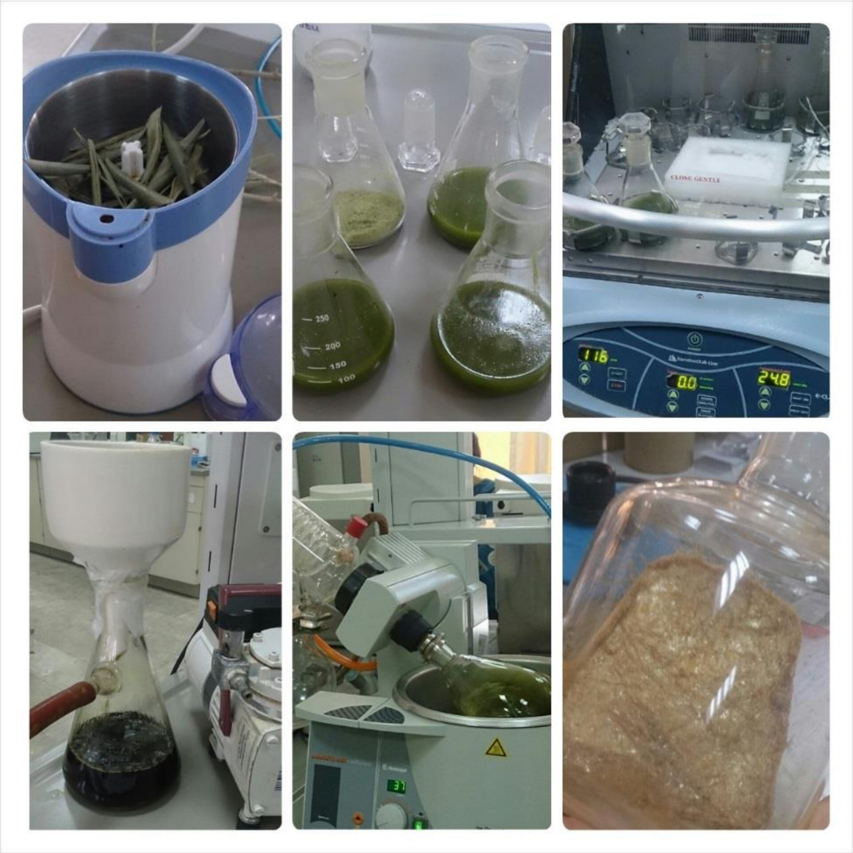

Figure S1. The extraction procedure of olive leave

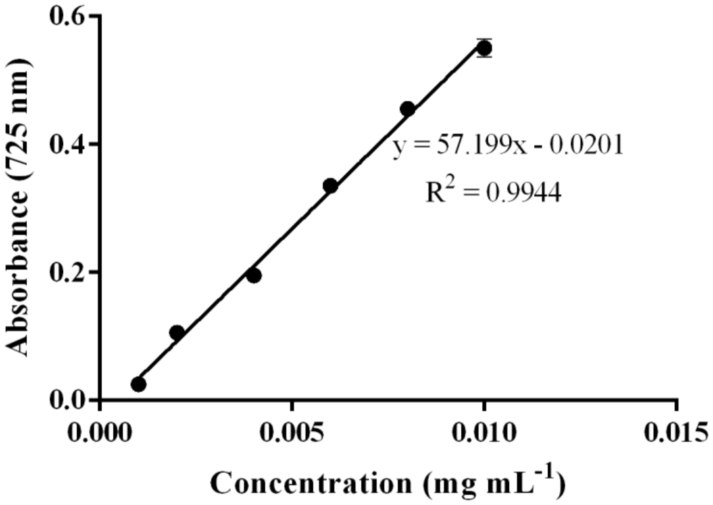

Figure S2. Calibration curve for total phenol content as gallic acid equivalent

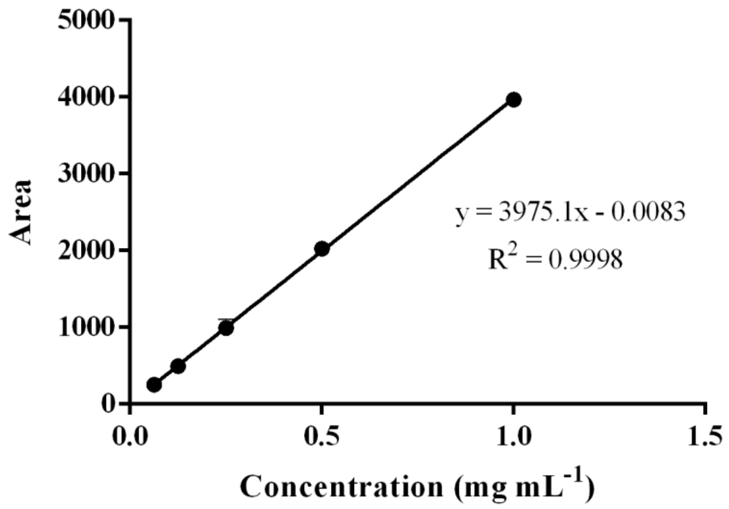

Figure S3. Calibration curve of oleuropein.

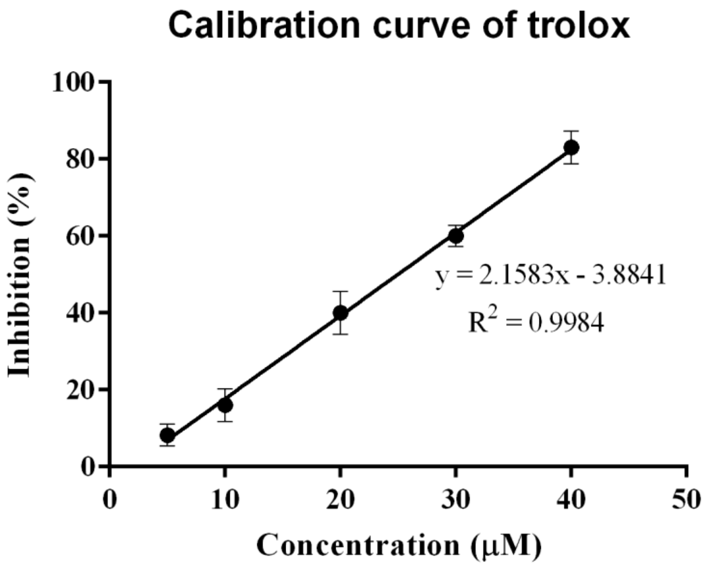

Figure S4. Calibration curve for total antioxidant capacity as mmol TEAC/g OLE

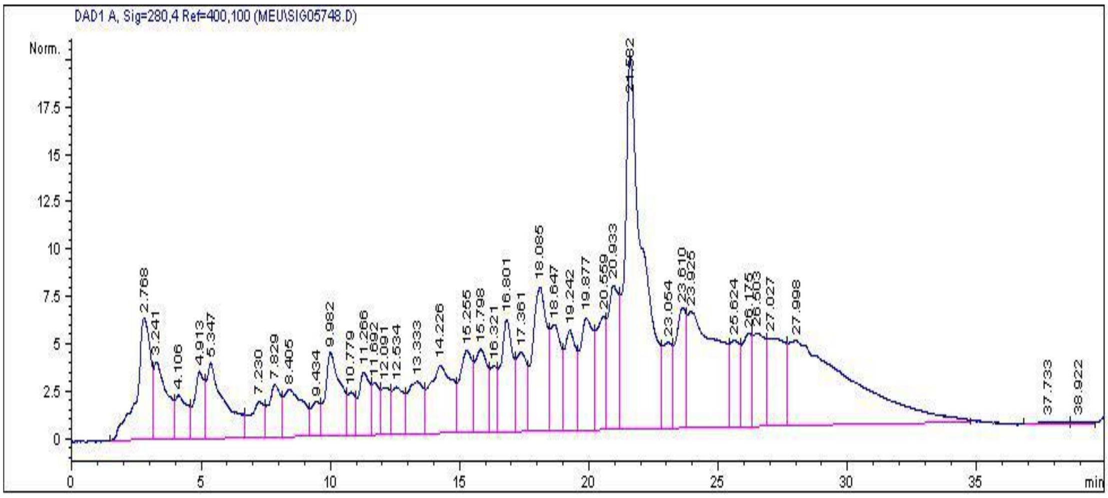

Figure S5. HPLC chromatogram of olive leaf extract

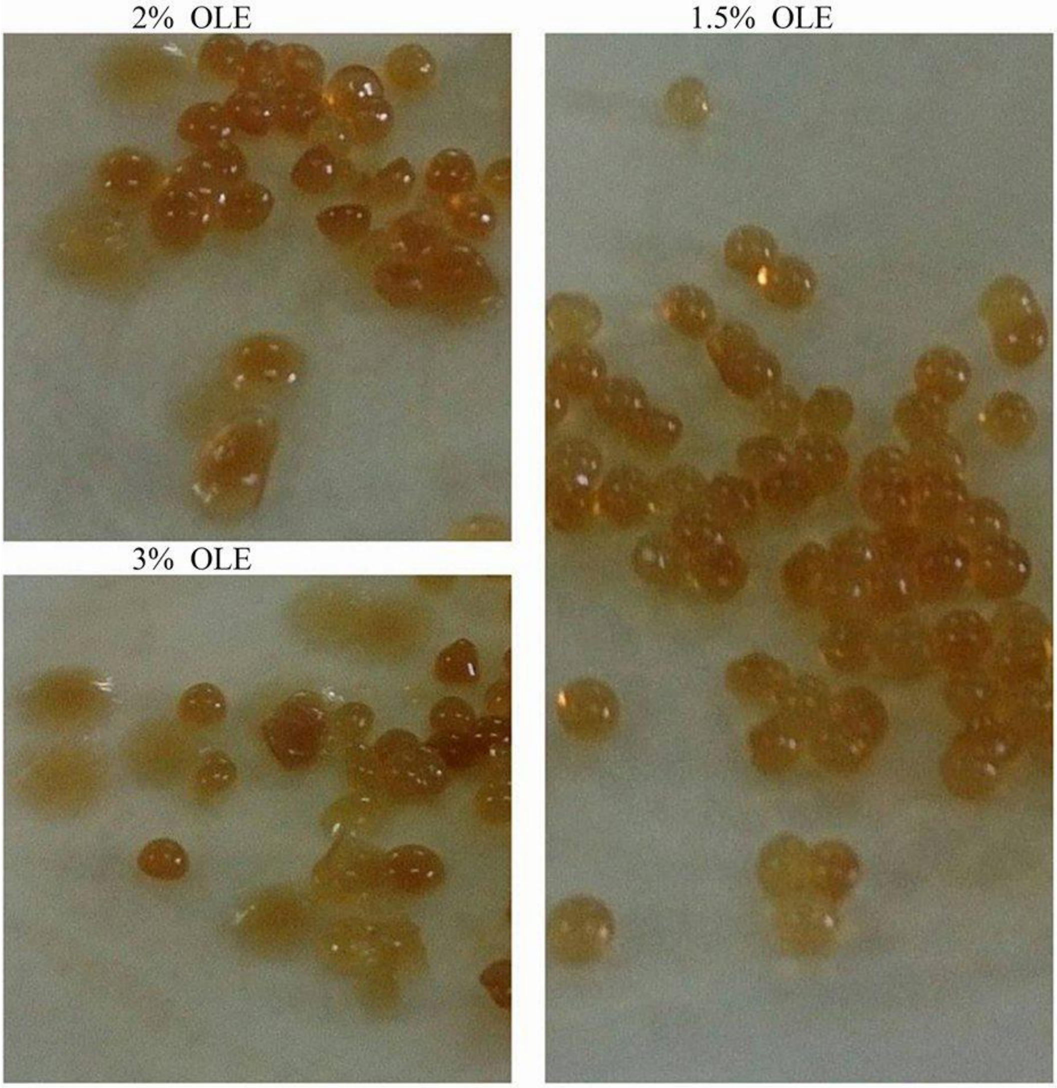

Figure S6. The effect of increasing OLE concentration on capsule formation.

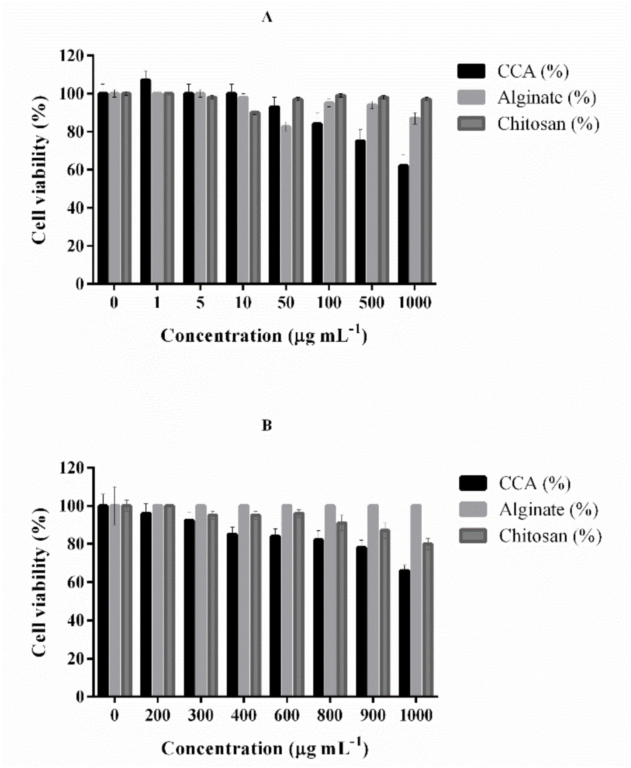

Figure S7. The cytotoxic effect of various concentrations of CCA, alginate and chitosan against A549 (**A**) and MCF-7 (**B**)

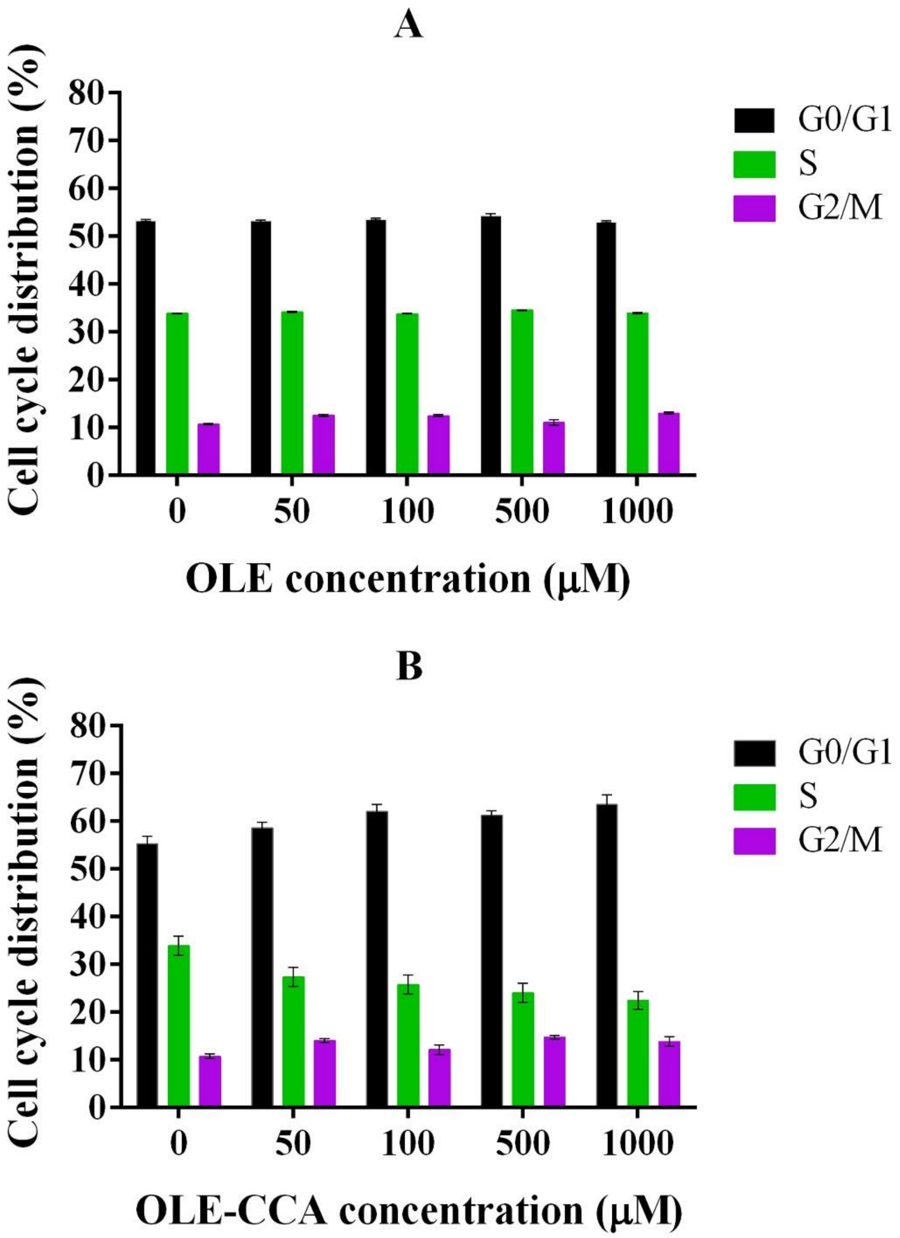

Figure S8. The effect of OLE (**A**) and OLE-CCA (**B**) on cell cycle arrest in MCF-7 cells
